# Combining donor-derived cell-free DNA and donor specific antibody testing as non-invasive biomarkers for rejection in kidney transplantation

**DOI:** 10.1038/s41598-022-19017-7

**Published:** 2022-09-05

**Authors:** Bogdan Obrișcă, Maria Butiu, Lena Sibulesky, Ramasamy Bakthavatsalam, Kelly D. Smith, Idoia Gimferrer, Paul Warner, Gener Ismail, Nicolae Leca

**Affiliations:** 1 Department of Nephrology, Fundeni Clinical Institute, Bucharest, Romania; 2 Division of Nephrology, University of Washington, Seattle, WA USA; 3 Division of Transplant Surgery, University of Washington, Seattle, WA USA; 4 Department of Laboratory Medicine and Pathology, University of Washington, Seattle, WA USA; 5 Bloodworks Northwest, Immunogenetics/HLA Laboratory, Seattle, WA USA

**Keywords:** Nephrology, Transplant immunology

## Abstract

Donor specific anti-HLA antibodies (DSA) and donor-derived cell-free DNA (dd-cfDNA) have lead to substantial progress in the non-invasive monitoring of the renal allograft by being able to detect or rule out subclinical rejection and guide immunosuppressive changes. In this study we sought to analyze the clinical, de novo DSA (*dn*DSA) and histological determinants of dd-cfDNA levels. The study included a cohort of stable renal function kidney transplant (KT) recipients who underwent anti-HLA *dn*DSA and dd-cfDNA testing between September 2017-December 2019. Statistical models were constructed to detect association with predictors of dd-cfDNA levels and other clinical characteristics. 171 renal allograft recipients were tested for dd-cfDNA and *dn*DSA at a median 1.06 years posttransplant (IQR: 0.37–4.63). Median dd-cfDNA was 0.25% (IQR: 0.19–0.51), 18.7% of patients having a dd-cfDNA ≥ 1%. In a multivariate linear regression model the presence of *dn*DSA MFI ≥ 2500 was the best independent determinant of dd-cfDNA level (p < 0.001). Among patients tested, 54 had concurrent dd-cfDNA determination at the time of an allograft biopsy. dd-cfDNA had an AUC of 0.82 (95% CI 0.69–0.91; p < 0.001) and of 0.96 (95% CI 0.87–0.99) to discriminate any rejection and ABMR, respectively. After multivariate adjustment, the models that included ABMR (*R* = 0.82, *R*^2^ = 0.67, p < 0.001), or ptc (*R* = 0.79, *R*^2^ = 0.63, p < 0.001) showed the best correlation with dd-cfDNA level. We are confirming a strong association of dd-cfDNA with *dn*DSA and underlying alloimmune-mediated injury in renal allograft recipients in a cohort of patients with unsuspecting clinical characteristics for rejection and excellent allograft function. Our findings support the need for noninvasive biomarker surveillance in KT recipients and we propose that dd-cfDNA may complement *dn*DSA screening.

## Introduction

Antibody-mediated rejection (ABMR) is the leading immunological cause of graft loss in kidney transplant recipients^1^. The death-censored 10-year allograft failure rates in USA are approximately 20–25% for deceased-donor and 15–16% for living-donor recipients^2^. The most recent ANZDATA registry report showed that up to 17% of graft failures within 1 year are attributable to acute rejection episodes, while up to 63% of graft failures beyond 1 year are due to chronic allograft nephropathy likely associated with late alloimmune injury^3^. In the past decade, several studies outlined the dominant role of the alloimmune-mediated allograft injury and de novo DSA (*dn*DSA) formation as determinants of long-term graft survival^1,4^.

Anti-HLA *dn*DSA formation, that occurs in up to 30% of renal allograft recipients, is the main pathogenic event correlated with late rejection and allograft failure^5^. Given that not all patients with *dn*DSA have ABMR^5^, allograft biopsy has remained the gold standard for diagnosis of rejection^6,7^. Nonetheless, the current clinical surveillance of graft function by means of serum creatinine and proteinuria measurements triggers a late diagnostic allograft biopsy from a pathogenic standpoint associated with prevalent chronic injury^8^. A recent study showed that a *dn*DSA-based surveillance protocol biopsy approach identified subclinical ABMR in 25 out of 102 patients^9^. In these patients, early identification and treatment of subclinical ABMR using *dn*DSA monitoring significantly improved allograft outcomes, compared to patients with clinical ABMR that underwent an indication biopsy due to a rise in serum creatinine^9^. Accordingly, there is an unmet need in kidney transplantation to identify non-invasive biomarkers that would detect earlier alloimmune-mediated pathogenic events and allow for earlier therapeutic interventions prior to substantial irreversible damage^8,10,11^.

Donor-derived cell-free DNA (dd-cfDNA) emerged as a candidate biomarker for detection of graft injury, particularly endothelial injury as seen in ABMR, but even more so for absence of injury with a high negative predictive value, in part due to the overall low prevalence of rejection in kidney transplant population^10,12^. Currently there is still a substantial variability in the timing of dd-cfDNA determination post-transplantantion and no standard has been established^13–18^. This aspect might bring a substantial limitation to the interpretation of these results given the overall short half-life time of this biomarker^12^.

Given that dd-cfDNA might be a more dynamic indicator of allograft health and earlier indicator of injury compared to other clinical variables, more attention should be paid to dd-cfDNA as a continuous variable rather than a cut-off based one. A recent study showed that there is a time-dependent increase in dd-cfDNA fraction over 5 years in stable kidney transplant recipients from 0.8 to 2.1%, which might bring into question if a cut-off based approach is suitable for all situations^19^. Accordingly, we sought to further analyze the clinical and histological determinants of dd-cfDNA and to characterize their associated magnitude in increase in absolute dd-cfDNA fraction level. In addition, we undertook an in-depth analysis of the diagnostic performance of dd-cfDNA for ABMR, in relation to other measures of graft function.

## Material and methods

### Study design and population

The study design has been previously reported^20^. Briefly, this is a cross-sectional study conducted at the University of Washington Medical Center kidney transplant program. Data was collected via electronic medical record review of patients (n = 171) who underwent *dn*DSA surveillance and dd-cfDNA testing as part of their standard of care between September 2017 and December 2019. Patients with multiorgan transplants or retransplantation, those with less than 3 months post-transplant follow-up or preformed DSA were excluded. This study was done with the approval of the Institutional Review Board of the University of Washington (STUDY00009002) and all participants provided written informed consent. All methods were conducted in compliance with the Declaration of Helsinki and the relevant guidelines and regulations.

### HLA antibodies identification

The cohort of patients enrolled in this study followed our institutional standards of *dn*DSA monitoring. *dn*DSAs were measured at 3, 6, 12 months, at anniversary visits or for cause at the time of biopsy. *dn*DSA screening was performed by Luminex technology (FLEXMAP 3D platform) using single-antigen beads (One Lambda, CA). *dn*DSAs were reported at a mean fluorescence intensity (MFI) cut-off of 500. Our center uses a MFI ≥ 2500 for reporting unacceptable antigens and reports DSA with MFI < 2500 as weak positive. The HLA antigens considered for *dn*DSA analysis were: A, B, C, DR, DQ, DP. The value of the calculated panel-reactive antibody (cPRA) used was the closest to transplantation and computed using the OPTN (Organ Procurement and Transplantation Network) procedures^21^. Briefly, cPRA is calculated based on the HLA frequencies among kidney donors in the United States and represents the percentage of organ donors that express at least one of the HLA antigens deemed unacceptable for a specific recipient based on the presence of preformed anti-HLA antibodies against: A, B, Bw, C, DR, DR51/52/53 and DQB1^22^.

### Clinical assessment and donor-derived cell-free DNA assay

The clinical assessment consisted of demographic data (age, gender), transplant characteristics (time from transplant, type of transplant, induction and maintenance immunosuppression), presence, type and MFI of *dn*DSA, renal function (serum creatinine and estimated glomerular filtration rate, eGFR), proteinuria and renal biopsy indication if applicable, as well as pathological diagnoses.

Measurement of dd-cfDNA was done as described in the DART trial^23^ using targeted next-generation sequencing assay. This method utilizes 266 single-nucleotide polymorphisms to quantify the dd-cfDNA without the need to separately genotype the donor and recipient (Allosure test, CareDx Inc.)^23^. The result was expressed as fraction of total cell-free DNA (cfDNA) with a quantifiable range of 0.1–16%. For the purpose of this analysis, dd-cfDNA was evaluated as both a continuous variable and a categorical variable with different thresholds used for sample classification as positive or negative. Dd-cfDNA testing was done for cause, once in each patient, in context of graft dysfunction, development of *dn*DSA or at the time of graft biopsy.

### Renal allograft biopsy assessment

Clinically indicated biopsies were performed in cases of renal dysfunction or elevated *dn*DSAs. Pathological diagnoses were made according to the 2017 Banff Kidney Rejection Classification^7^. The patients were stratified on whether they had ABMR (acute, active ABMR or chronic, active ABMR), T-cell mediated rejection (TCMR) or both. In addition, each individual pathological component of ABMR and TCMR were scored separately in order to test their association with dd-cfDNA level. The kidney allograft biopsies were evaluated by a renal pathologist who was unaware of the result of dd-cfDNA level measurement.

### Statistical analysis

Continuous variables were expressed as either mean ± standard deviation or median (interquartile range: 25th–75th percentiles), according to their distribution, and categorical variables as percentages. Differences between groups were assessed in case of continuous variables by Student *t* test, Mann–Whitney test, one-way ANOVA or Kruskal–Wallis test, according to the distribution of dependent variables and the level of independent variable, and in case of categorical variables by Pearson χ^2^ test or Fisher’s exact test. In addition, differences of dd-cfDNA levels according to severity of histologic lesions were assessed by Mann–Whitney or Kruskal–Wallis testing, as appropriate. Spearman’s rank correlation test was used to assess the relationship between *dn*DSA MFI and absolute dd-cfDNA level. Performance characteristics of clinical variables to discriminate rejection and ABMR from other types of graft injury were assessed by receiver-operating characteristic curve (area under the curve, AUC). To assess the predictive ability of different measures of allograft function for rejection/ABMR identification, we calculated sensitivity, specificity, positive (PPV) and negative (NPV) predictive values, positive likelihood ratio (LR+), negative likelihood ratio (LR−) and accuracy, with corresponding 95% confidence intervals (95% CI). Linear regression models were performed to evaluate determinants of absolute dd-cfDNA level. In addition, univariate and multivariate logistic regression analysis were performed to identify predictors of ABMR. In all analyses, p values are two-tailed and all p values less than 0.05 were considered statistically significant.

Statistical analyses were performed using the SPSS program (SPSS version 20, Chicago, IL), GraphPad Prism version 9.3.1 (1992–2021 GraphPad Software, LLC) and MedCalc® Statistical Software version 20.014 (MedCalc Software Ltd, Ostend, Belgium; https://www.medcalc.org; 2021).

### Human transplantation statement

Study was performed in accordance with international standards. All patients underwent kidney transplantation at the University of Washington Medical Center Kidney Transplant Program. NO organs/tissues were procured from prisoners.

## Results

### Study population

Between September 2017 and December 2019, 171 renal allograft recipients were tested for dd-cfDNA and *dn*DSA at a median 1.06 years posttransplant (IQR: 0.37–4.63). Median dd-cfDNA was 0.25% (IQR: 0.19–0.51), 25.7% of all patients having a dd-cfDNA level over 0.5%, while 18.7% had a dd-cfDNA level over 1%. A total of 43 patients (25.1%) had positive *dn*DSA values. Five patients (2.9%) had weak isolated HLA class I *dn*DSAs with a median MFI of 2000 (IQR: 1500–2150), while 38 patients (22.2%) developed HLA class II *dn*DSAs with median MFI of 12,800 (IQR: 2875–22,900), three of them having both HLA class I and II *dn*DSAs.

### Clinical predictors of high levels of donor-derived cell-free DNA

When analyzing baseline patients’ characteristics (Table 1), a high plasma dd-cfDNA level (> 1%) was significantly associated with increased transplant vintage (4.16 vs. 0.95 years; p = 0.019) and with development of *dn*DSAs against HLA class II antigens. There were no significant differences between the groups of patients with low versus high dd-cfDNA levels in terms of other recipient, transplant or immunosuppression characteristics. Patients with HLA class II *dn*DSA and those with mixed HLA class I/II *dn*DSA had higher dd-cfDNA [median 0.62%, (IQR:0.19–2.7) and 3.6%, (IQR: 0.85–3.6), respectively] than those with isolated HLA class I *dn*DSA (median 0.3%, IQR: 0.22–0.74%) or negative *dn*DSA (median 0.22% (IQR: 0.17–0.37) (p < 0.001). Similar associations between *dn*DSAs and dd-cfDNA level were observed when using a lower cut-off of 0.5% for considering a high dd-cfDNA (Supplemental Table 1).

**Table 1:** Baseline patient characteristics according to the level of dd-cfDNA.

Variable	Low dd-cfDNA (< 1%)	High dd-cfDNA (≥ 1%)	P value
Number of patients	139	32	
**Demographic data**			
Age (years)	53 ± 15	49 ± 13	0.19
Gender (n, % males)	83 (59.7%)	14 (43.8%)	0.1
Race, n (%)			
White	79 (56.8%)	18 (56.2%)	0.18
Hispanic	10 (7.2%)	7 (21.9%)	
Asian	24 (17.3%)	3 (9.4%)	
Black/African American	17 (12.2%)	3 (9.4%)	
Native Hawaiian or Other Pacific Islander/American Indian/Alaska Native	9 (6.5%)	1 (3.1%)	
Time posttransplant to dd-cfDNA determination (years)	0.95 (IQR: 0.29–3.84)	4.16 (IQR: 0.66–5.94)	0.019
Time posttransplant, n (%)			
< 6 months	53 (38.1%)	7 (21.9%)	**0.03**
6–12 months	20 (14.4%)	3 (9.4%)	
1–5 years	38 (27.3%)	10 (31.2%)	
5–10 years	15 (10.8%)	10 (31.2%)	
> 10 years	13 (9.4%)	2 (6.2%)	
Type of donor, n (%)			
Deceased-donor	103 (74.1%)	21 (65.6%)	0.33
Living-donor	36 (25.9%)	11 (34.4%)	
**Immunosuppression characteristics**			
Induction immunosuppression, n (%)			
Thymoglobulin	106 (86.9%)	26 (89.7%)	> 0.99
Basiliximab	16 (13.1%)	3 (10.3%)	
Maintenance immunosuppression, n (%)			
Tacrolimus	132 (95%)	31 (96.9%)	0.2
Cyclosporine	2 (1.4%)	0 (0%)	
Sirolimus	0 (0%)	1 (3.1%)	
Belatacept	2 (1.4%)	0 (0%)	
Mycophenolic acid	118 (81.8%)	26 (81.2%)	0.62
Azathioprine	2 (1.4%)	1 (3.1%)	
Leflunomide	1 (0.7%)	1 (3.1%)	
Prednisone	137 (98.6%)	31 (96.9%)	0.46
Immunosuppression dosage/level at induction and at dd-cfDNA measurement			
Thymoglobulin (total dose, mg)	197 ± 123	202 ± 123	0.91
FK level (ng/ml)	6.97 ± 3.01	6.87 ± 3.76	0.89
Mycophenolic acid (mg/day)	720 (IQR: 360–720)	720 (IQR: 360–990)	0.57
**Laboratory data**			
Serum creatinine at dd-cfDNA measurement (mg/dl)	1.54 ± 0.52	1.41 ± 0.51	0.18
Serum creatinine at last follow-up (mg/dl)	1.56 ± 0.67	1.54 ± 0.76	0.35
eGFR at dd-cfDNA measurement (ml/min/1.73 m^2^)	50 ± 20	57 ± 26	0.15
eGFR at last follow-up (ml/min/1.73 m^2^)	51 ± 21	55 ± 27	0.48
UPCR at dd-cfDNAmeasurement (g/g)	0.2 (IQR: 0.1–0.39)	0.2 (IQR: 0.1–0.92)	0.79
UPCR at last-follow-up (g/g)	0.2 (IQR: 0.1–0.42)	0.2 (IQR: 0.1–0.58)	0.4
Calculated panel reactive antibody			
< 20%	112 (80.6%)	28 (87.5%)	0.36
20–50%	8 (5.8%)	0 (0%)	
> 50%	19 (13.7%)	4 (12.5%)	
dd-cfDNA level (median %, IQR)	0.21 (IQR: 0.17–0.31)	2.1 (IQR: 1.3–2.95)	**< 0.001**
***dn*DSA characteristics**			
Patients with *dn*DSAs, n (%)			
No *dn*DSA	114 (82%)	14 (43.8%)	**< 0.001**
HLA class I *dn*DSA	4 (2.9%)	1 (3.1%)	
HLA class II *dn*DSA	20 (14.4%)	15 (46.9%)	
HLA class I + II *dn*DSA	1 (0.7%)	2 (6.2%)	
*dn*DSA MFI at dd-cfDNA measurement (highest), n (%)			
Negative	114 (82%)	14 (43.8%)	**< 0.001**
< 2500	9 (6.5%)	2 (6.2%)	
≥ 2500	16 (11.5%)	16 (50%)	
*dn*DSA MFI (median, IQR)	2900 (IQR: 1900–20,800)	13,200 (IQR: 3500–22,900)	0.2

dd-cfDNA, donor-derived cell-free DNA; *dn*DSA, de novo donor-specific antibody; MFI, mean fluorescence intensity; eGFR, estimated glomerular filtration rate; UPCR, urine protein:creatinine ratio.

Additionally, the *dn*DSA MFI were correlated with absolute dd-cfDNA levels (*r* = 0.36; p < 0.001). In patients with *dn*DSA MFI ≥ 2500, median dd-cfDNA level was 0.96% (IQR: 0.28–2.85), compared to 0.28% (IQR: 0.19–0.34) in those with *dn*DSA MFI < 2500 and 0.22% (IQR: 0.17–0.37) in *dn*DSA negative patients (p < 0.001).

In the univariate linear regression analysis, among all clinical variables, the presence of *dn*DSA MFI ≥ 2500 was the best predictor of the dd-cfDNA fraction determining an increase in the absolute level by approximately 1.27% (β coefficient: 1.27; 95% CI 0.94–1.59; *R* = 0.51, *R*^2^ = 0.26, *p* < 0.001). The level of protein:creatinine ratio only weakly correlated with dd-cfDNA level (coefficient of correlation, *R* = 0.17 and coefficient of determination, *R*^2^ = 0.03; β coefficient: 0.15, 95% CI 0.02–0.27, p = 0.02), while the serum creatinine did not correlate with dd-cfDNA level (Table 2). In the multivariate linear regression model (*R* = 0.57, *R*^2^ = 0.32, *p* < 0.001), the presence of *dn*DSA MFI ≥ 2500 was identified as the independent determinant of absolute dd-cfDNA level (β coefficient: 1.27; 95% CI 0.94–1.6; *p* < 0.001). Nonetheless, among the classical measures of allograft function, the level of protein:creatinine ratio independently predicted the fraction of dd-cfDNA, albeit with a significantly lower impact compared to *dn*DSA MFI, determining an increase by only 0.16% for each 1 g/g of creatinine (Table 2).

**Table 2:** Linear regression to evaluate determinants of absolute dd-cfDNA values.

Variables	Unadjusted			Adjusted^a^		
	β coefficient (95% CI)	Std. error	p-value	β coefficient (95% CI)	Std. error	p-value
Age (for each 1 year)	− 0.11 (− 0.2, − 0.002)	0.005	0.02	− 0.006 (− 0.01, 0.003)	0.005	0.18
Gender (M vs. F)	− 0.08 (− 0.37, 0.21)	0.14	0.58	− 0.001 (− 0.26, 0.26)	0.13	0.99
Ethnicity (Caucasian vs. other)	− 0.05 (− 0.34, 0.23)	0.14	0.71	0.21 (− 0.05, 0.48)	0.13	0.12
Type of donor (deceased-donor vs. living-donor)	− 0.08 (− 0.41, 0.23)	0.16	0.59	0.14 (− 0.17, 0.47)	0.16	0.36
Time from Tx to dd-cfDNA measurement (for each 1 year)	0.009 (− 0.18, 0.3)	0.01	0.5	− 0.007 (− 0.03, 0.02)	0.01	0.59
Serum creatinine (for each 1 mg/dl)	− 0.17 (− 0.45, 0.1)	0.14	0.21	− 0.26 (− 0.52, 0.003)	0.13	0.053
UPCR (for each 1 g/g)	0.15 (0.02, 0.27)	0.06	0.02	0.16 (0.04, 0.28)	0.06	0.008
FK level (ng/mL)	0.01 (− 0.3, 0.06)	0.02	0.55	0.01 (0.04, 0.28)	0.02	0.43
*dn*DSA MFI < 2500 (vs. negative *dn*DSA)	0.11 (− 0.39, 0.62)	0.26	0.66	0.16 (− 0.37, 0.69)	0.27	0.55
*dn*DSA MFI ≥ 2500 (vs. negative *dn*DSA)	1.27 (0.94, 1.59)	0.16	< 0.001	1.27 (0.94, 1.6)	0.16	< 0.001

dd-cfDNA, donor-derived cell-free DNA; *dn*DSA, de novo donor-specific antibody; MFI, mean fluorescence intensity; Tx, transplant; UPCR, urine protein:creatinine ratio; M, male; F, female.

^a^After multivariate adjustment for age, gender, race, type of transplant, serum creatinine, UPCR, FK level, time to dd-cfDNA determination and dnDSA MFI.

### Dd-cfDNA accurately discriminates ABMR

Of the 171 patients, 54 had concurrent dd-cfDNA determination at the time of a clinically indicated allograft biopsy. The allograft biopsy was triggered in the majority of cases by evidence of allograft dysfunction (increased serum creatinine in 68.5% of patients and/or proteinuria in 40.7% of cases) and/or by development of *dn*DSA (35.1%). In addition, three patients underwent an allograft biopsy as part of surveillance protocols. There were 12 patients with ABMR (of whom one patient with a surveillance biopsy and negative *dn*DSA), 12 patients with TCMR and 6 with mixed lesions consistent with both ABMR and TCMR.

**Figure 1:**
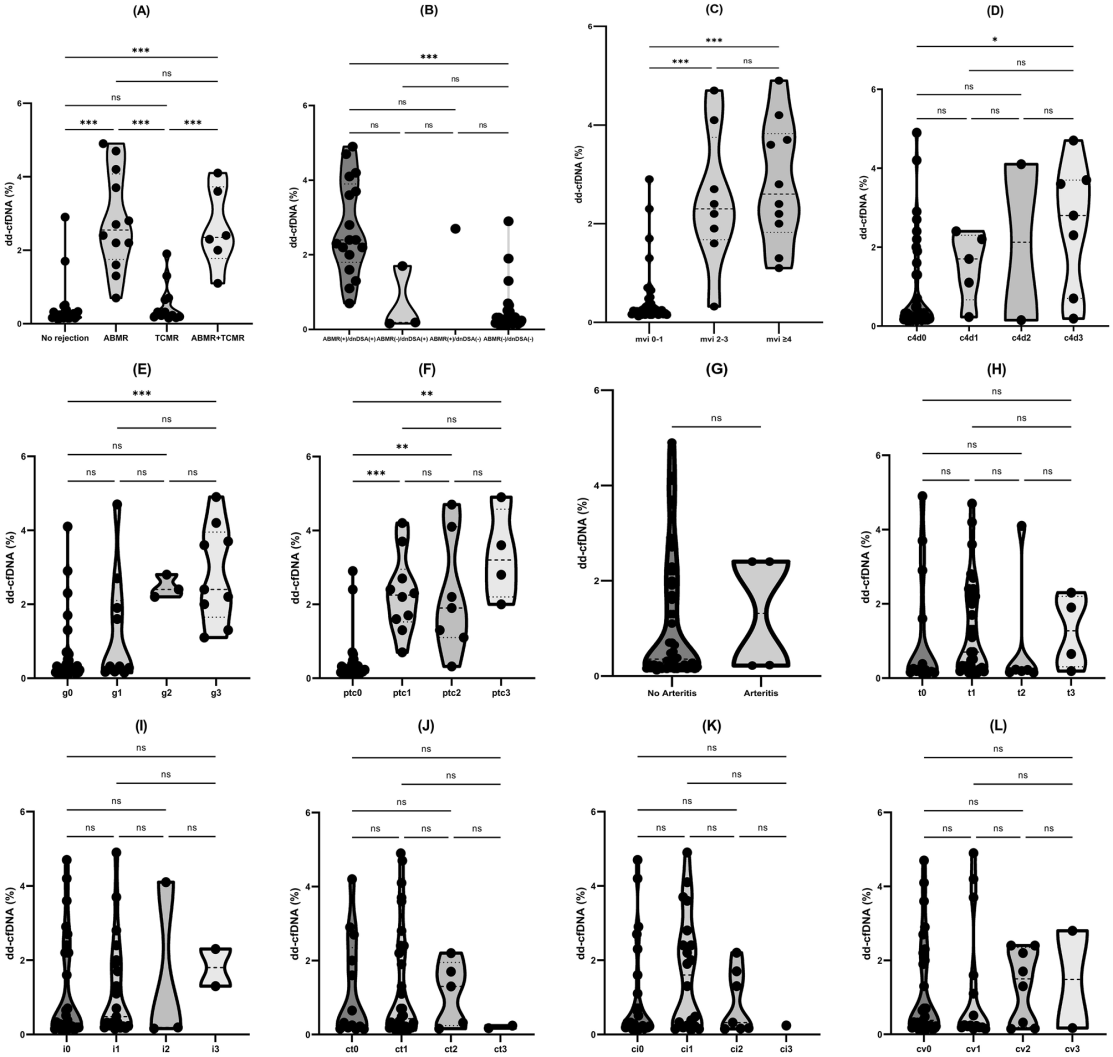
The absolute level of dd-cfDNA and its correlation with rejection type and individual pathological lesions (ABMR, antibody-mediated rejection; TCMR, T-cell mediated rejection; *dn*DSA, donor-specific antibodies; mvi, microvascular inflammation; g, glomerulitis; ptc, peritubular capillaritis; t, tubulitis; i, interstitial inflammation; ct, tubular atrophy; ci, interstitial fibrosis; cv, arteriosclerosis. Differences of dd-cfDNA levels were assessed by Mann–Whitney or Kruskal–Wallis testing, as appropriate; p-values are reported as following: *p between 0.01 and 0.05, **p between 0.001 and 0.01, ***p < 0.001).

**Figure 2:**
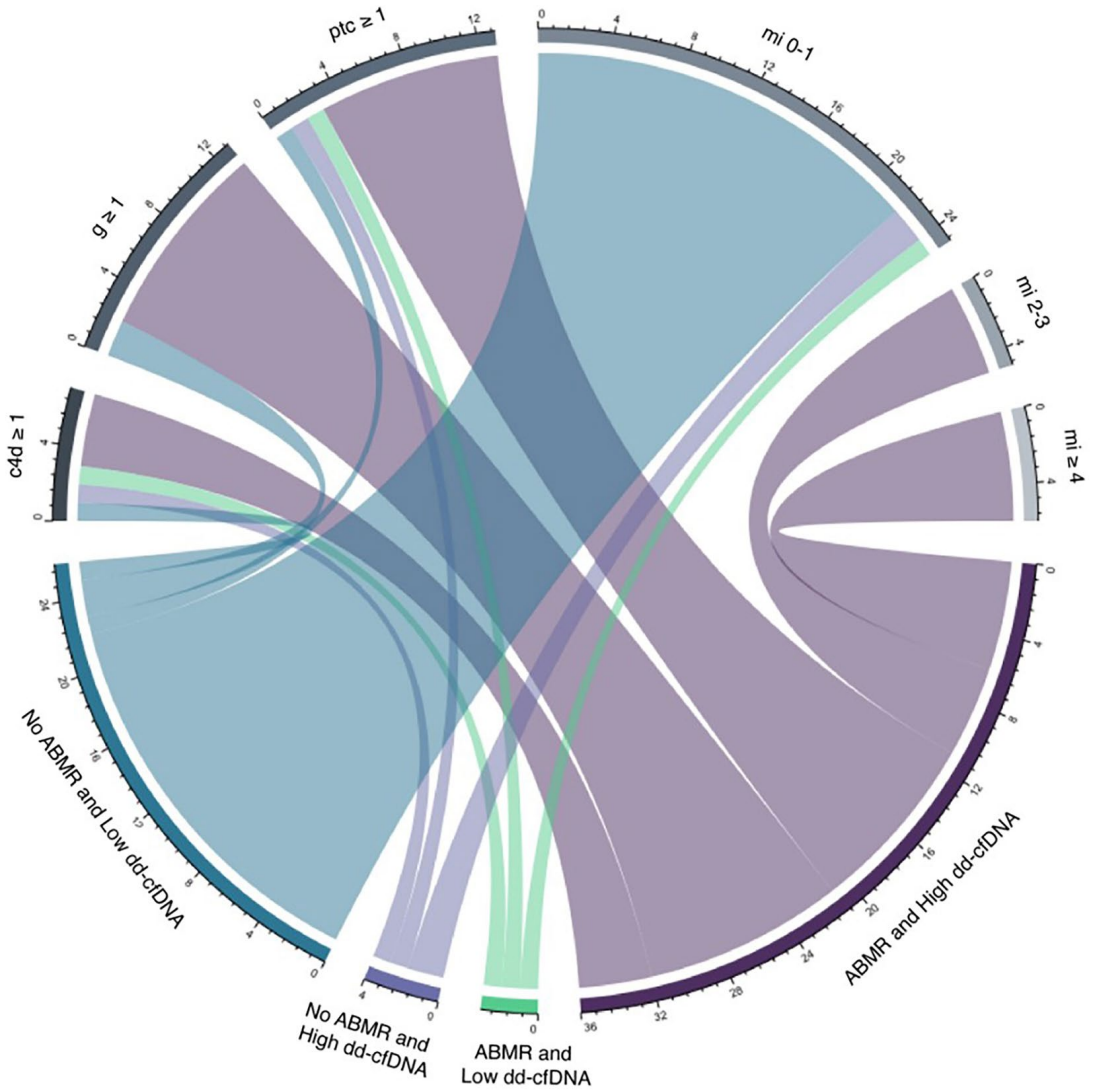
Distribution of histological lesions according to presence of ABMR and dd-cfDNA level. (The numbers in the upper part of the figure (elementary lesion sector) indicate the number of patients with each lesion. The numbers in the lower part indicate the total number of elementary lesions for each category of ABMR presence and dd-cfDNA level).

As shown in Table 3, those with allograft biopsy features consistent with ABMR had a dd-cfDNA level above 1%, except for one patient with ABMR alone that had a dd-cfDNA of 0.7%. Moreover, 76.2% of those with high dd-cfDNA had ABMR and *dn*DSA. Median level of dd-cfDNA was 2.55% (IQR: 1.75–4.07) in patients with ABMR and 2.35% (IQR: 1.77–3.72) in those with mixed ABMR/TCMR, compared to 0.27% (IQR: 0.18–0.68) in patients with TCMR alone and 0.2% (IQR: 0.15–0.32) in those without rejection (p < 0.001) (Fig. 1A and Supplemental Table 2). As shown in Fig. 2, apart from the overt association with ABMR, the dd-cfDNA fraction correlates with the severity of individuals lesions associated with alloimmune injury and it may identify patients that do not fulfill all the criteria for ABMR, but may have significant underlying activity. Of the 18 patients with microvascular inflammation (MVI = g + ptc ≥ 2) on kidney transplant biopsy, 2 patients did not meet the criteria for ABMR and had a dd-cfDNA level of 0.32% and 1.9%, while all patients with MVI that met criteria for ABMR had a dd-cfDNA over 1% (Fig. 2). In addition, dd-cfDNA may identify patients with incipient ABMR as seen in the two patients with high *dn*DSA MFI that had minimal MVI (C4d3, ptc1, g0) and dd-cfDNA level of 0.7% and 2.3%, respectively (Fig. 2).

**Table 3:** Baseline patient characteristics according to the level of dd-cfDNA (biopsy cohort).

Variable	Low dd-cfDNA (< 1%)	High dd-cfDNA (≥ 1%)	p value
Number of patients	33	21	
**Demographic data**			
Age (years)	54 ± 17	49 ± 13	0.27
Gender (n, % males)	23 (69.7%)	10 (47.6%)	0.1
Race, n (%)			
White	17 (51.5%)	10 (47.6%)	0.78
Other	16 (48.5%)	11 (52.4%)	
Time posttransplant to dd-cfDNA determination (years)	0.58 (IQR: 0.23–1.73)	4.63 (IQR: 1.1–5.98)	**0.001**
Time posttransplant, n (%)			
< 6 months	14 (42.4%)	3 (14.3%)	**< 0.001**
6–12 months	8 (24.2%)	1 (4.8%)	
1–5 years	8 (24.2%)	8 (38.1%)	
5–10 years	0 (0%)	9 (42.9%)	
> 10 years	3 (9.1%)	0 (0%)	
Type of donor, n (%)			
Deceased-donor	28 (84.8%)	14 (66.7%)	0.18
Living-donor	5 (15.2%)	7 (33.3%)	
**Immunosuppression characteristics**			
Induction immunosuppression, n (%)			
Thymoglobulin	25 (75.7%)	17 (81%%)	> 0.99
Basiliximab	5 (15.1%)	3 (14.3%)	
Maintenance immunosuppression, n (%)			
Tacrolimus	32 (97%)	21 (100%)	> 0.99
Cyclosporine	1 (3%)	0 (0%)	
Mycophenolic acid	29 (87.9%)	18 (85.7%)	> 0.99
Prednisone	33 (100%)	21 (100%)	> 0.99
Immunosuppression dosage/level at induction and at dd-cfDNA measurement			
Thymoglobulin (total dose, mg)	249 ± 67	277 ± 86	0.37
FK level (ng/ml)	7.65 ± 2.84	6.43 ± 2.21	0.08
Mycophenolic acid (mg/day)	720 (IQR: 360–720)	720 (IQR: 720–1080)	0.08
**Laboratory data**			
Serum creatinine at dd-cfDNA measurement (mg/dl)	1.89 ± 0.57	1.36 ± 0.5	**0.001**
Serum creatinine at last follow-up (mg/dl)	1.89 ± 0.71	1.55 ± 0.87	**0.006**
eGFR at dd-cfDNA measurement (ml/min/1.73 m^2^)	41 ± 21	60 ± 25	**0.008**
eGFR at last follow-up (ml/min/1.73 m^2^)	42 ± 21	58 ± 27	**0.04**
UPCR at dd-cfDNA measurement (g/g)	0.2 (IQR: 0.16–0.8)	0.3 (IQR: 0.1–1.45)	0.9
UPCR at last-follow-up (g/g)	0.3 (IQR: 0.1–2.55)	0.5 (IQR: 0.1–1.6)	0.75
Calculated panel reactive antibody			
< 20%	30 (90.9%)	19 (90.5%)	0.65
20–50%	1 (3%)	0 (0%)	
> 50%	2 (6.1%)	2 (9.5%)	
dd-cfDNA level (median %, IQR)	0.22 (IQR: 0.16–0.32)	2.4 (IQR: 1.8–3.65)	**< 0.001**
***dn*DSA characteristics**			
Patients with d*n*DSAs, n (%)			
No *dn*DSAs	30 (90.9%)	4 (19%)	**< 0.001**
HLA class I *dn*DSAs	0 (0%)	0 (0%)	
HLA class II *dn*DSAs	3 (9.1%)	14 (66.7%)	
HLA class I + II *dn*DSAs	0 (0%)	3 (14.3%)	
*dn*DSA MFI at dd-cfDNA measurement (highest), n (%)			
Negative	30 (90.9%)	4 (19%)	**< 0.001**
< 2500	0 (0%)	1 (4.8%)	
≥ 2500	3 (9.1%)	16 (76.2%)	
**Biopsy findings**			
Any rejection, n (%)	11 (33.3%)	19 (90.5%)	**< 0.001**
Type of rejection, n (%)			
No rejection	22 (66.7%)	2 (9.5%)	**< 0.001**
ABMR	1 (3%)	11 (52.4%)	
TCMR	10 (30.3%)	2 (9.5%)	
ABMR + TCMR	0 (0%)	6 (28.6%)	
Distribution of patients according to the presence of ABMR and *dn*DSAs			
* dn*DSA and ABMR	1 (3%)	16 (76.2%)	**< 0.001**
* dn*DSA without ABMR	2 (6.1%)	1 (4.8%)	
ABMR without *dn*DSA	0 (0%)	1 (4.8%)	
No *dn*DSA, No ABMR	30 (90.9%)	3 (14.3%)	
Individual lesions, n of pts (%)			
Cd4 staining ≥ 1	4 (12.1%)	10 (47.6%)	**0.004**
Glomerulitis (g) ≥ 1	6 (18.2%)	16 (76.2%)	**< 0.001**
Peritubular capillaritis (ptc) ≥ 1	2 (6.1%)	19 (90.5%)	**< 0.001**
Microvascular inflammation (g + ptc)			
0–1	32 (97%)	4 (19%)	**< 0.001**
2–3	1 (3%)	7 (33.3%)	
≥ 4	0 (0%)	10 (47.6%)	
Presence of transplant glomerulopathy	1 (3%)	9 (42.9%)	**< 0.001**
Presence of arteritis	2 (6.1%)	2 (9.5%)	0.63
Tubulitis (t) ≥ 2	7 (21.1%)	3 (14.3%)	0.72
Interstitial inflammation (i) ≥ 2	2 (6.1%)	3 (14.3%)	0.36
Tubular atrophy (ct) ≥ 2	4 (12.1%)	3 (14.3%)	> 0.99
Interstitial fibrosis (ci) ≥ 2	5 (15.2%)	3 (14.3%)	> 0.99
Arteriosclerosis (cv) ≥ 2	4 (12.1%)	6 (28.6%)	0.49

dd-cfDNA, donor-derived cell-free DNA; *dn*DSA, de novo donor-specific antibody; MFI, mean fluorescence intensity; ABMR, antibody-mediated rejection; TCMR, T-cell mediated rejection; eGFR, estimated glomerular filtration rate; UPCR, urine protein:creatinine ratio.

**Figure 3:**
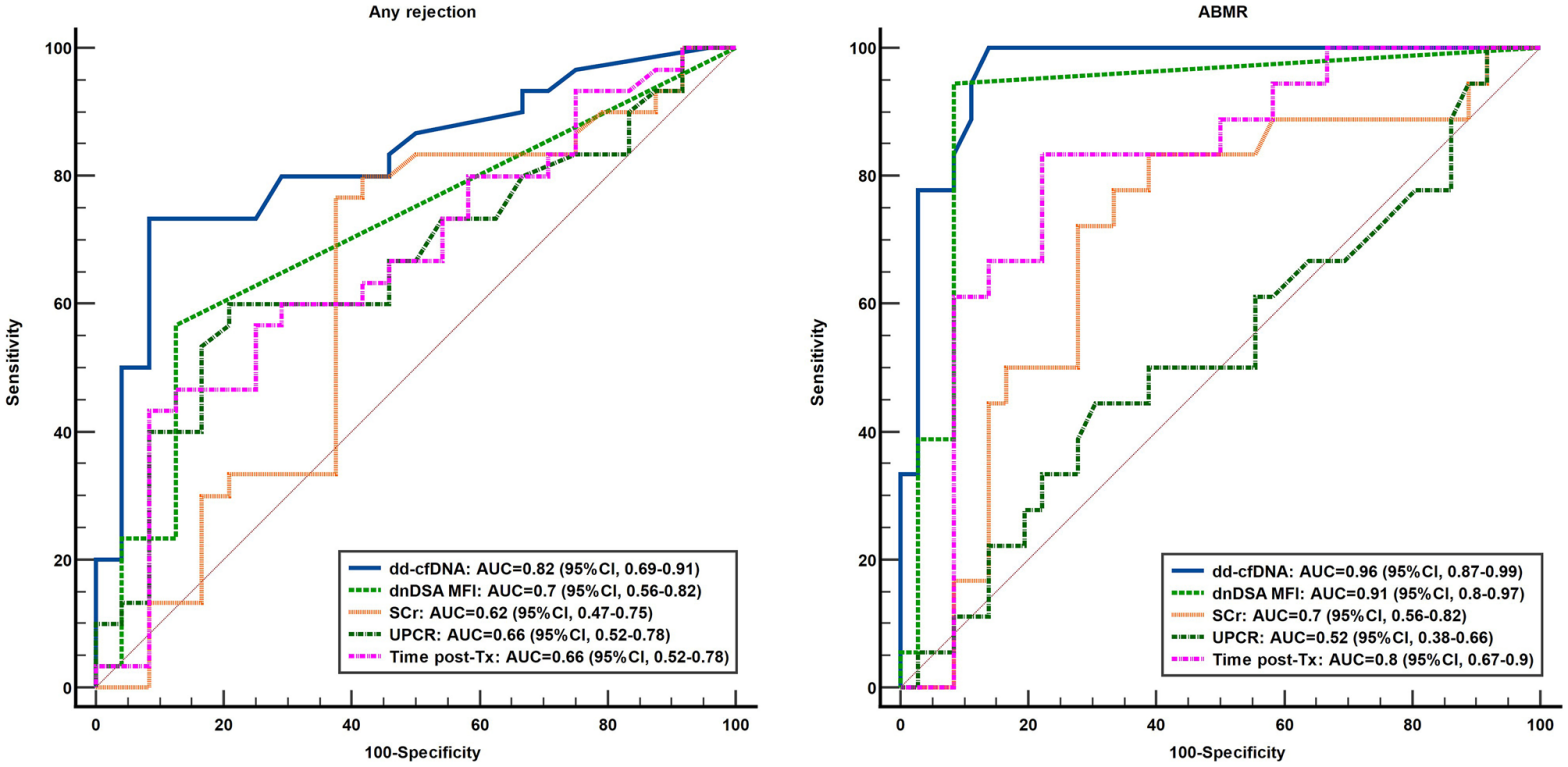
dd-cfDNA discriminates any rejection and ABMR with better performance characteristics compared to other measures of graft function (*dn*DSA, donor-specific antibodies, UPCR, urine protein:creatinine ratio; SCr, serum creatinine; Tx, transplant; AUC, area under the curve).

Compared to other measures of graft injury, dd-cfDNA had the best performance characteristics with an AUC of 0.82 (95% CI 0.69–0.91; p < 0.001) to discriminate any rejection and an AUC of 0.96 (95% CI 0.87–0.99; p < 0.001) to discriminate ABMR (Fig. 3). In addition, a cut-off of 1% had the best predictive capacity for ABMR with a sensitivity of 94.4% (95% CI 72–99%), PPV of 80.9% (95% CI, 62–91%) and a diagnostic accuracy of 90.7% (95% CI 79–96%) (Table 4). Nonetheless, given that an elevated dd-cfDNA level may also reflect non-rejection type of injuries, in terms of the utility as a surveillance biomarker, the dd-cfDNA has an excellent negative predictive value being able to rule out graft injury with an accuracy superior to other measures of graft function [NPV of 96.9% (95% CI 82–99%) and LR- of 0.06 (95% CI 0.01–0.4)] (Table 4). Moreover, in multivariate logistic regression analysis, an elevated dd-cfDNA level was more strongly associated with ABMR compared to *dn*DSA MFI (Supplemental Table 3). In addition, when combining the dd-cfDNA and *dn*DSA MFI the diagnostic accuracy significantly increased compared to each individual variable, while retaining the excellent negative predictive capacity (Table 4).

**Table 4:** Performance characteristics of clinical variables to discriminate any rejection and antibody-mediated rejection.

Variable	Sensitivity (95% CI)	Specificity (95% CI)	PPV (95% CI)	NPV (95% CI)	LR+ (95% CI)	LR− (95% CI)	Accuracy (95% CI)
**dd-cfDNA ≥ 0.5%**							
Any rejection	73.3% (54–87%)	87.5% (67–97%)	88% (71–95%)	72.4% (58–82%)	5.87 (1.9–17.2)	0.3 (0.1–0.5)	79.6% (66–89%)
ABMR	100% (81–100%)	80.5% (63–91%)	72% (56–83%)	100%	5.1 (2.6–10)	0	87% (75–94%)
**dd-cfDNA ≥ 0.74%**							
Any rejection	63.3% (43–80%)	91.6% (73–98%)	90.4% (71–97%)	66.6% (55–76%)	7.6 (1.9–29.4)	0.4 (0.2–0.6)	75% (62–86%)
ABMR	94.4% (72–99%)	88.9% (74–96%)	80.9% (62–91%)	96.9% (82–99%)	8.5 (3.3–21.5)	0.06 (0.01–0.4)	90.7% (79–96%)
**dd-cfDNA ≥ 1%**							
Any rejection	63.3% (43–80%)	91.6% (73–98%)	90.4% (71–97%)	66.6% (55–76%)	7.6 (1.9–29.4)	0.4 (0.2–0.6)	75% (62–86%)
ABMR	94.4% (72–99%)	88.9% (74–96%)	80.9% (62–91%)	96.9% (82–99%)	8.5 (3.3–21.5)	0.06 (0.01–0.4)	90.7% (79–96%)
***dn*DSA MFI ≥ 2500**							
Any rejection	53.3% (34–71%)	87.5% (67–97%)	84.2% (63–94%)	60% (49–69%)	4.27 (1.4–12.9)	0.5 (0.3–0.8)	68.5% (54–80%)
ABMR	88.9% (65–98%)	91.6% (77–98%)	84.2% (64–94%)	94.3% (81–98%)	10.67 (3.5–31.9)	0.12 (0.03–0.4)	90.7% (79–96%)
**dd-cfDNA ≥ 1% and *dn*DSA MFI ≥ 2500**							
Any rejection	50% (31–68%)	96% (78–99%)	93.7% (68–99%)	60.5% (51–68%)	12 (1.7–84.5)	0.5 (0.3–0.7)	70.3% (56–82%)
ABMR	83.3% (58–96)	97.2% (85–99%)	93.7% (68–99%)	92.1% (80–97%	30 (4.3–209)	0.17 (0.06–0.4)	92.6% (82–97%)
**SCr ≥ 1.2 mg/dl**							
Any rejection	70% (50–85%)	20.8% (7–42%)	52.5% (44–60%)	35.7% (17–59%)	0.88 (0.6–1.2)	1.44 (0.5–3.7)	48.1% (34–62%)
ABMR	55.5% (30–78%)	16.6% (6–32%)	25% (17–34%)	42.8% (23–64%)	0.6 (0.4–1.03)	2.67 (1.09–6.5)	29.6% (17–43%)
**UPCR > 0.2 g/g**							
Any rejection	73.3% (54–87%)	23.8% (8–47%)	57.9% (49–65%)	38.4% (19–62%)	0.96 (0.7–1.3)	1.12 (0.4–2.9)	52.9% (38–67%)
ABMR	61.1% (35–82%)	18.1% (6–35%)	28.9% (21–37%)	46.1% (25–68%)	0.75 (0.5–1.12)	2.14 (0.8–5.4)	33.3% (20–47%)

dd-cfDNA, donor-derived cell-free DNA; *dn*DSA, de novo donor-specific antibody; ABMR, antibody-mediated rejection; PPV, positive-predictive value; NPV, negative predictive value; UPCR, urine protein:creatinine ratio; SCr, serum creatinine; LR+, positive likelihood ratio; LR−, negative likelihood ratio; CI, confidence interval.

Among the 24 patients with no rejection, 19 had no significant pathological lesions (89% with low dd-cfDNA). One patient had recurrent IgA nephropathy (dd-cfDNA, 0.28%), 2 had BK virus nephropathy (dd-cfDNA, 0.24% and 0.48%), one had acute pyelonephritis (dd-cfDNA, 0.33%) and one had acute tubular necrosis (dd-cfDNA, 0.19%). Another 4 patients had concomitant rejection (TCMR or ABMR) and recurrent glomerular disorders (IgA nephropathy or focal and segmental glomerulosclerosis), all with low dd-cfDNA in the absence of ABMR.

### Donor-derived cell-free DNA associates with Banff elementary lesions of microvascular inflammation

We then analyzed the dd-cfDNA distribution according to Banff elementary lesions (Table 3, Fig. 1). Among the determinant histological lesions for a high dd-cfDNA level, we observed a graded response with the intensity of C4d staining, glomerulitis score and peritubular capillaritis score (Fig. 1D–F). Additionally, MVI score (MVI = g + ptc) showed a strong correlation with dd-cfDNA level (Fig. 1C). We did not identify any correlation between active or chronic tubulointerstitial lesions lesions and dd-cfDNA (Fig. 1H–K). Despite that dd-cfDNA showed consistent association with lesions typical of MVI and endothelial injury, we did not find any association with endotheliitis, probably due to the small number of patients that had this lesion on allograft biopsy (n = 4) (Fig. 1G).

Table 5 details the models of linear regression analysis for the biopsy cohort. In univariate analysis, the presence of *dn*DSA with MFI ≥ 2500, antibody-mediated rejection, intense C4d staining (C4d3), at least moderate glomerulitis and any peritubular capillaritis scores were among the determinants of absolute dd-cfDNA level. By contrary, serum creatinine had a negative correlation with dd-cfDNA given that those with an elevated dd-cfDNA had an overall better graft function (Tables 3 and 5), while proteinuria had a non-significant impact on absolute dd-cfDNA level. After multivariate adjustment, the models that included ABMR or peritubular capillaritis showed the best correlation with dd-cfDNA level. The model that accounted for ABMR explained 67% of the variability of dd-cfDNA level (*R* = 0.82, *R*^2^ = 0.67, *p* < 0.001), while the model that accounted for peritubular capillaritis explains 63% of the variability of dd-cfDNA level (*R* = 0.79, *R*^2^ = 0.63, *p* < 0.001) outperforming the models that incorporated C4d staining or glomerulitis score. Specifically, the presence of *dn*DSA MFI ≥ 2500 determines an increase in absolute dd-cfDNA level by 1.87%, while the presence of ABMR determines an increase by 2.2–2.6%. In addition, the severity of peritubular capillaritis independently determines an increase in absolute dd-cfDNA level ranging 1.84–2.91% (Table 5). There was no association with tubulitis, interstitial inflammation or chronic lesions scores (data not shown).

**Table 5:** Linear regression to evaluate determinants of absolute dd-cfDNA values.

	Unadjusted			Adjusted^a^		
	β coefficient (95% CI)	Std. error	p-value	β coefficient (95% CI)	Std. error	p-value
**Intercept**	0.54 (0.18, 0.89)	0.17	0.003	0.96 (− 0.5; 2.44)	0.73	0.19
+ *dn*DSA MFI ≥ 2500	1.88 (1.28, 2.47)	0.29	< 0.001	1.87 (1.17; 2.57)	0.35	< 0.001
**Intercept**	2.75 (1.71, 3.8)	0.51	< 0.001	3.59 (1.79; 5.39)	0.89	< 0.001
+ SCr (for 1 mg/dl)	− 0.92 (− 1.5, − 0.33)	0.29	0.003	− 1.006 (− 1.68; − 0.33)	0.33	0.004
**Intercept**	1.13 (0.7, 1.57)	0.21	< 0.001	2.62 (0.75; 4.49)	0.93	0.007
+ UPCR (for 1 g/g)	0.06 (− 0.14, 0.27)	0.1	0.53	− 0.08 (− 0.32; 0.16)	0.2	0.5
**Intercept**	0.41 (0.05, 0.76)	0.17	0.02	0.92 (− 0.24; 2.09)	0.58	0.12
+ ABMR	2.37 (1.75, 2.99)	0.31	< 0.001	2.6 (1.89; 3.32)	0.35	< 0.001
+ TCMR	0.11 (− 0.5, 0.73)	0.31	0.7	0.17 (− 0.47; 0.83)	0.32	0.59
+ ABMR/TCMR	2.17 (1.37, 2.97)	0.39	< 0.001	2.24 (1.4; 3.08)	0.41	< 0.001
**Intercept**	0.87 (0.47, 1.28)	0.2	< 0.001	1.73 (− 0.009; 3.48)	0.86	0.05
+ C4d1	0.64 (− 0.56, 1.85)	0.6	0.28	0.67 (− 0.59; 1.93)	0.63	0.29
+ C4d2	1.24 (− 0.59, 3.09)	0.92	0.18	0.37 (0–1.99; 2.73)	1.17	0.75
+ C4d3	1.69 (0.64, 2.73)	0.52	0.002	1.65 (0.51; 2.8)	0.56	0.006
**Intercept**	0.62 (0.23, 1.03)	0.19	0.002	1.6 (0.16; 3.04)	0.71	0.03
+ g1	0.61 (− 0.19, 1.41)	0.39	0.13	0.83 (− 0.17; 1.83)	0.5	0.1
+ g2	1.84 (0.5, 3.18)	0.66	0.008	2.21 (0.7; 3.72)	0.75	0.005
+ g3	2.2 (1.36, 3.03)	0.41	< 0.001	2.58 (1.59; 3.58)	0.49	< 0.001
**Intercept**	0.4 (0.08, 0.72)	0.15	0.01	0.79 (− 0.46; 2.05)	0.62	0.21
+ ptc1	1.87 (1.21, 2.53)	0.33	< 0.001	1.84 (1.14; 2.55)	0.35	< 0.001
+ ptc2	1.82 (1.06, 2.59)	0.38	< 0.001	2.14 (1.25; 3.03)	0.44	< 0.001
+ ptc3	2.92 (1.95, 3.89)	0.48	< 0.001	2.91 (1.86; 3.96)	0.52	< 0.001

dd-cfDNA, donor-derived cell-free DNA; *dn*DSA, de novo donor-specific antibody; MFI, mean fluorescence intensity; AMR, antibody-mediated rejection; TCMR, T-cell mediated rejection; UPCR, urine protein:creatinine ratio; SCr, serum creatinine; g, glomerulitis; ptc, peritubular capillaritis.

^a^After multivariate adjustment for age, race, gender, type of transplant ,FK level and time post-transplant.

## Discussion

In this study, we have further shown that the presence of *dn*DSA with high MFI (≥ 2500) against HLA class II antigens are clinically significant and the most important determinant of absolute dd-cfDNA fraction level, while there was no significant correlation with either serum creatinine or proteinuria (evaluated in routine clinical monitoring). Moreover, we confirmed that combining an elevated dd-cfDNA level with *dn*DSA MFI determines a diagnostic accuracy for the presence of ABMR superior to either independent variable alone and significantly outperforms the classical measures of graft function. In addition, among the most important predictors of an absolute increase in dd-cfDNA fraction level are lesions associated with microvascular inflammation (mostly peritubular capillaritis), suggesting that it may be a useful biomarker reflecting the severity of endothelial injury.

In our study, of the 171 renal allograft recipients, 43 patients (25.1%) had positive *dn*DSA, worth mentioning this does not represent the *dn*DSA incidence but rather the percentage of patients with *dn*DSA in the cross-sectional population surveyed during the study period. In our previous analysis, an elevated dd-cfDNA level (≥ 1%) was significantly associated with an 11-fold higher chance for the presence of *dn*DSA with MFI ≥ 2500^20^. Nonetheless, although the dd-cfDNA fraction threshold of 1% has been validated in several studies, there is a concern that there is a time-dependent variability in the dd-cfDNA fraction^24,25^. A recent study of 303 clinically stable kidney transplant recipients identified a steady increase in dd-cfDNA fraction from 0.8 to 2.1% in the first 5 years post-transplant^19^. In our study, although the median time for dd-cfDNA testing was approximately 1 year post-transplantation, 25% of the study cohort had a dd-cfDNA determination after 4.6 years post-transplant. This aspect prompted us to undertake a different approach in terms of defining the clinical utility of this putative biomarked in kidney transplant recipients, by considering dd-cfDNA both as a threshold and as a continuous variable. Accordingly, we have further confirmed our initial observations that the presence of *dn*DNAs with a MFI ≥ 2500 is of clinical significance since it was independently associated with an absolute increase in dd-cfDNA fraction by approximately 1.27%. By comparison, the presence of *dn*DSA with MFI < 2500 determined an increase in dd-cfDNA fraction by only 0.16% (not statistically significant).

These findings have several implications. First, our analysis outlines the limitations of routine clinical monitoring of alloimmune-mediated graft injury by means of measuring serum creatinine or proteinuria. In our cohort, proteinuria only weakly correlated and could explain only 3% of the variability in the dd-cfDNA level, while serum creatinine had a negative correlation with dd-cfDNA level and, in fact, those with an elevated dd-cfDNA (over 1%) had an overall better serum creatinine values (1.41 ± 0.51) compared to those with low dd-cfDNA (1.54 ± 0.52). Contrary, only the development of *dn*DSA with a high MFI (≥ 2500) were associated with the absolute increase in dd-cfDNA fraction.However, after multivariate adjustment, the linear regression model explained only 32% of the variability in dd-cfDNA level. This could be explained by the lack of allograft biopsies in all patients and the inability to rule out other types of graft injury. In addition, a substantial proportion of patients with elevated dd-cfDNA (approximately 44%) were negative for anti-HLA antibodies and, in such patients, we cannot confidently exclude a non-HLA antibody mediated-graft injury. If we would account for this hypothesis, the explanation for the variability level of dd-cfDNA would increase to over 70%.

To our knowledge, only one study addressed the relationship between dd-cfDNA and developing *dn*DSA^26^. In this study, among the 79 patients with early T-cell mediated rejection, those with high dd-cfDNA (> 0.5%) had a 28-fold higher risk of developing *dn*DSA, emphasizing the risk posed by early graft injury on subsequent alloimmune response^26^. It has been hypothesized that the dd-cfDNA may be a more sensitive marker of ABMR than *dn*DSA. If the newly formed *dn*DSA are all bound to allograft endothelium, it may trigger injury signal long before it is detected in the blood. However our study has not substantiated this relationship at lower level of *dn*DSA.

Performance characteristics of dd-cfDNA to identify ABMR are better than those reported in other trials (AUC, 0.96, compared to 0.87, in the DART trial^23^ and to 0.82, in the study reported by Huang et al.^16^). Despite that several cut-offs for an elevated dd-cfDNA level have been evaluated (0.5%^26^, 0.74%^16^, 0.88%^27^ or 1%^17,23^), we have identified the best performance for a cut-off of 1%. Other studies have shown conflicting results with regards to the superiority of dd-cfDNA over serum creatinine in the discrimination of patients with or without ABMR^23,27^. In our cohort, the AUC for serum creatinine to discriminate ABMR was 0.7, similar to the study of Gielis et al. (AUC, 0.64) and superior to the DART trial (AUC, 0.57)^23,27^. However, in the previous studies an adequate assessment of dd-cfDNA’s diagnostic performance in relation to other measures of graft function was not thoroughly undertaken. In our study the best predictor of any rejection or ABMR was dd-cfDNA fraction, with similar, but better diagnostic performance than *dn*DSA MFI and far superior than that of proteinuria or serum creatinine. In our data, the biopsies were more likely driven by graft dysfunction in the low dd-cfDNA group with higher creatinine levels versus the biomarker driven biopsies in the elevated dd-cfDNA cohort (creatinine 1.89 vs. 1.36; p < 0.001). Despite this, two-thirds of the patients in the low dd-cfDNA did not have rejection versus 90% in the high dd-cfDNA cohort. This finding, further outlines the lack of specificity of allograft dysfunction in relation to rejection and the importance of dd-cfDNA as an useful biomarker in detecting subclinical rejection. Moreover, combining the dd-cfDNA and *dn*DSA MFI determined an excellent diagnostic accuracy especially for ABMR of 93%, while retaining an excellent NPV for the absence of graft injury.

In addition to confirming the strong association with ABMR, we have shown that there is a graded response of dd-cfDNA level according to the severity of microvascular lesions. As compared to C4d staining or glomerulitis, the multivariate linear regression model incorporating the peritubular capillaritis score showed the highest correlation with absolute dd-cfDNA level. Although a correlation between absolute levels of dd-cfDNA and microvascular inflammation lesions has been previously shown^18,27^, this is the first time that the true impact of pathological lesions on an elevated dd-cfDNA was assessed in a multivariate linear regression analysis, after adjusting for potential confounders (such as recipients, transplant or immunosuppression characteristics). Our data is supported by the results from the Trifecta study in which dd-cfDNA level correlated with the molecular phenotype of kidney transplant biopsies (ABMR-related and peritubular capillaritis molecular classifiers)^13^. However, although our study did not show a strong association with TCMR alone, it is worth mentioning that the Trifecta study identified a reasonably strong correlation with active TCMR^13^. Our results may have been limited by the overall small number of patients with an allograft biopsy as 16.6% of patients with TCMR alone (n = 2) had an elevated dd-cfDNA fraction.

Previous work on this subject noted that dd-cfDNA testing may not always be rejection specific, but an expression of cellular injury and turnover^28^. As such, elevated levels have been detected in other clinical scenarios: delayed-graft function, recurrence of glomerular disease, BK virus nephropathy, infection or acute tubular necrosis^23,27^. Notably, in our allograft-biopsy cohort, we did not identify increased dd-cfDNA levels in patients with other types of graft injury, in the absence of concomitant ABMR. The reasons for these discordant results might be related to differences of study cohorts particularly in relation to the severity of injury from renal impairment perspective, the timing of evaluation in relation to transplantation procedure and overall small number of cases.

The reason for the strong association with MVI might derive from the close proximity of endothelial cells with the circulation^28^. As such, dd-cfDNA testing may have potential to detect injury prior to detectable *dn*DSAs and possibly even beyond current classification of ABMR which may not be sensitive enough for the early changes of MVI in absence of detectable *dn*DSAs. Additionally, dd-cfDNA may allow the detection of ABMR-mediated by non-HLA antibodies as shown by one patient in our cohort with ABMR in the absence of detectable *dn*DSA. The overall good allograft function in this study cohort (and even better in patients with elevated dd-cfDNA) further supports the utility of dd-cfDNA to early identify the subclinical alloimmune-mediated injury.

A substantial limitation of our study is the *dn*DSA-based surveillance of our cohort. Future dd-cfDNA-based surveillance may allow better definition of the association of dd-cfDNA with rejection especially in the early stages of injury, possibly independent of *dn*DSA. Although a dedicated prospective trial to evaluate a monitoring schedule posttransplantation for dd-cfDNA is clearly needed, from a cost-efectiveness perspective it has been proposed that a weekly testing in the first month, followed by monthly testing for 6 months and then quarterly testing thereafter may be appropriate. Furthermore, future prospective interventional trials, ideally with surveillance biopsies, may allow studying potential improvements in allograft outcomes allowed by earlier interventions prompted by elevations in dd-cfDNA. A second limitation to our study is the absence of an allograft biopsy in all patients which may limit the generalizability of our results. In addition, given that the majority were for cause biopsies, there may be a concern for a selection bias. However, the biopsy cohort had a heterogeneity of histologic lesions, including non-rejection type of injury. Nonetheless, this is one of the largest cohorts of patients tested concomitantly for *dn*DSAs and dd-cfDNA with a more in-depth analysis of both the predictors of dd-cfDNA fraction and its diagnostic performance for alloimmune-mediated injury.

In conclusion, in this study we have confirmed a strong association of dd-cfDNA with alloimmune-mediated injury in renal-allograft recipients. Thus, we propose that dd-cfDNA should become part of rejection surveillance methods, at least as a complementary method along with *dn*DSA screening.

## Supplementary Information

Supplementary Tables.

## Data Availability

All data generated or analysed during this study are included in this published article [and its supplementary information files].
