# Supplementary material for: Combining donor-derived cell-free DNA and donor specific antibody testing as non-invasive biomarkers for rejection in kidney transplantation
Source: Sci Rep. 2022 Sep 5;12:15061. doi: 10.1038/s41598-022-19017-7 (PMC9445050; doi:10.1038/s41598-022-19017-7)
Supplement: Supplementary file 1 — Supplementary Tables. [file 41598_2022_19017_MOESM1_ESM.docx]

**Supplemental Table 1. Baseline patient characteristics according to the presence of low versus high level of donor-derived cell-free DNA (cut-off 0.5%)**

| **Variable** | **Low dd-cfDNA (<0.5%)** | **High dd-cfDNA (≥0.5%)** | **P value** |
| --- | --- | --- | --- |
| **Number of patients** | 127 | 44 |  |
| ***Demographic data*** | | | |
| **Age (years)** | 53 ± 15 | 50 ± 14 | 0.18 |
| **Gender (n, % males)** | 77 (60.6%) | 20 (45.5%) | 0.08 |
| **Race, n (%)** |  |  |  |
| White | 73 (57.5%) | 24 (54.5%) | 0.37 |
| Mexican | 9 (7.1%) | 8 (18.2%) |  |
| Asian | 22 (17.3%) | 5 (11.4%) |  |
| Black/African American | 15 (11.8%) | 5 (11.4%) |  |
| Native Hawaiian or Other Pacific Islander/ American Indian/Alaska Native | 8 (6.3%) | 2 (4.5%) |  |
| **Time postransplant to ddcf-DNA determination (years)** | 0.98  (IQR:0.29-2.86) | 3.67  (IQR:0.44-5.94) | **0.035** |
| **Time posttransplant , n (%)** |  |  |  |
| <6 months | 49 (38.6%) | 11 (25%) | 0.06 |
| 6-12 months | 18 (14.2%) | 5 (11.4%) |  |
| 1-5 years | 35 (27.6%) | 13 (29.5%) |  |
| 5-10 years | 13 (10.2%) | 12 (27.3%) |  |
| >10 years | 12 (9.4%) | 3 (6.8%) |  |
| **Type of Tx, n (%)** |  |  |  |
| Cadaveric | 93 (73.2%) | 31 (70.5%) | 0.72 |
| Living | 34 (26.8%) | 13 (29.5%) |  |
| ***Immunosuppression characteristics*** | | | |
| **Induction immunosuppression, n (%)** |  |  |  |
| Thymoglobulin | 98 (77.1%) | 34 (77.2%) | >0.99 |
| Basiliximab | 14 (5.1%) | 5 (11.3%) |  |
| **Maintenance immunosuppression, n (%)** |  |  |  |
| Tacrolimus | 120 (94.5%) | 43 (97.7%) | 0.25 |
| Cyclosporine | 2 (1.6%) | 0 (0%) |  |
| Sirolimus | 0 (0%) | 1 (2.3%) |  |
| Belatacept | 2 (1.6%) | 0 (0%) |  |
| Mycophenolic acid | 107 (84.3%) | 37 (84.1%) | 0.27 |
| Azathioprine | 1 (0.8%) | 2 (4.5%) |  |
| Leflunomide | 1 (0.8%) | 1 (2.3%) |  |
| Prednisone | 125 (98.4%) | 43 (97.7%) | >0.99 |
| **Immunosuppression dosage/level at induction and at dd-cfDNA measurement** |  |  |  |
| Thymoglobulin (total dose, mg) | 198 ± 123 | 199 ± 121 | 0.88 |
| FK level (ng/ml) | 6.94 ± 3.05 | 6.97 ± 3.45 | 0.96 |
| Mycophenolic acid (mg/day) | 720 (IQR:360-720) | 720 (IQR:260-720) | 0.36 |
| ***Laboratory data*** | | | |
| **Serum Creatinine at dd-cfDNA measurement (mg/dl)** | 1.53 ± 0.52 | 1.48 ± 0.52 | 0.55 |
| **Serum Creatinine at last follow-up (mg/dl)** | 1.53 ± 0.57 | 1.65 ± 0.94 | 0.88 |
| **eGFR at dd-cfDNA measurement (ml/min/1.73m2)** | 51 ± 20 | 54 ± 24 | 0.5 |
| **eGFR at last follow-up (ml/min/1.73m2)** | 52 ± 21 | 52 ± 26 | 0.99 |
| **Urine Protein/Creatinine at ddcfDNA measurement** | 0.2  (IQR:0.1-0.39) | 0.2  (IQR:0.1-0.92) | 0.54 |
| **Urine Protein/Creatinine at last-follow-up** | 0.2  (IQR:0.1-0.4) | 0.2  (IQR:0.1-0.67) | 0.19 |
| **Calculated panel reactive antibody** |  |  |  |
| <20% | 104 (81.9%) | 36 (81.8%) | 0.99 |
| 20-50% | 6 (4.7%) | 2 (4.5%) |  |
| >50% | 17 (13.4%) | 6 (13.6%) |  |
| **dd-cfDNA level (median %, IQR)** | 0.19 (IQR:0.16-0.28) | 1.35 (IQR:0.87-2.67) | **<0.001** |
| ***dnDSA characteristics*** | | | |
| **Patients with dnDSAs, n (%)** |  |  |  |
| No dnDSAs | 106 (83.5%) | 22 (50%) | **<0.001** |
| Class I dnDSAs | 4 (3.1%) | 1 (2.3%) |  |
| Class II dnDSAs | 17 (13.4%) | 18 (40.9%) |  |
| Class I +II dnDSAs | 0 (0%) | 3 (6.8%) |  |
| **dnDSA titer at ddcfDNA measurement (highest), n (%)** |  |  |  |
| - Negative | 106 (83.5%) | 22 (50%) | **<0.001** |
| - <2500 | 9 (7.1%) | 2 (4.5%) |  |
| - >2500 | 12 (9.4%) | 20 (45.5%) |  |
| **dnDSA MFI titer (median, IQR)** | 2600 (IQR:1750-23550) | 13200 (IQR:7475-22450) | 0.1 |

Abbreviations: dd-cfDNA, donor-derived cell-free DNA; dnDSA, de novo donor-specific antibody; MFI, mean fluorescence intensity; Tx, transplant.

**Supplemental Table 2. Univariate analysis of the variables associated with rejection type.**

| **Variable** | **No rejection** | **ABMR** | **TCMR** | **Mixed (ABMR+TCMR)** | **P value** |
| --- | --- | --- | --- | --- | --- |
| **Number of patients** | 24 | 12 | 12 | 6 |  |
| ***Demographic data*** | | | | | |
| **Age (years)** | 51 ± 16 | 48 ± 11 | 58 ± 18 | 49 ± 17 | 0.36 |
| **Gender (n, % males)** | 15 (62.5%) | 8 (66.7%) | 7 (58.3%) | 3 (50%) | 0.91 |
| **Race, n (%)** |  |  |  |  | 0.91 |
| White | 13 (54.2%) | 6 (50%) | 5 (41.7%) | 3 (50%) |  |
| Other | 11 (45.8%) | 6 (50%) | 7 (58.3%) | 3 (50%) |  |
| **Time postransplant to ddcf-DNA determination (months)** | 8.2  (IQR:2.9-24.6) | 60.3  (IQR:26.3-83.1) | 6.6  (IQR:2.9-13.7) | 51.9  (IQR:7.1-65.3) | **0.003** |
| **Type of Tx, n (%)** |  |  |  |  | 0.2 |
| Cadaveric | 21 (87.5%) | 7 (58.3%) | 10 (83.3%) | 4 (66.7%) |  |
| Living | 3 (12.5%) | 5 (41.7%) | 2 (16.7%) | 2 (33.3%) |  |
| ***Immunosuppression characteristics*** | | | | | |
| **Induction immunosuppression, n (%)** |  |  |  |  | 0.16 |
| Thymoglobulin | 20 (83.3%) | 10 (83.3%) | 7 (58.3%) | 5 (83.3%) |  |
| Basiliximab | 2 (8.3%) | 2 (16.7%) | 4 (33.3%) | 0 (0%) |  |
| **Maintenance immunosuppression, n (%)** |  |  |  |  |  |
| Tacrolimus | 23 (95.8%) | 12 (100%) | 12 (100%) | 6 (100%) | 0.73 |
| Cyclosporine | 1 (4.2%) | 0 (0%) | 0 (0%) | 0 (0%) |  |
| Mycophenolic acid | 22 (91.7%) | 11 (91.7%) | 10 (83.3%) | 4 (66.7%) | 0.38 |
| Prednisone | 24 (100%) | 12 (100%) | 12 (100%) | 6 (100%) |  |
| **Immunosuppression dosage/level at induction and at dd-cfDNA measurement** |  |  |  |  |  |
| Thymoglobulin (total dose, mg) | 241 ± 75 | 307 ± 88 | 250 ± 47 | 265 ± 57 | 0.18 |
| FK level (ng/ml) | 7.65 ± 2.32 | 6 ± 1.69 | 7.8 ± 3.78 | 6.25 ± 2.33 | 0.2 |
| Mycophenolic acid (mg/day) | 720 ± 400 | 810 ± 379 | 510 ± 350 | 660 ± 576 | 0.31 |
| ***Laboratory data*** | | | | | |
| **Serum Creatinine at dd-cfDNA measurement (mg/dl)** | 1.83 ± 0.69 | 1.36 ± 0.45 | 1.77 ± 0.45 | 1.56 ± 0.53 | 0.13 |
| **Serum Creatinine at last follow-up (mg/dl)** | 1.68 ± 0.61 | 1.64 ± 0.95 | 2.07 ± 0.92 | 1.69 ± 0.84 | 0.23 |
| **eGFR at dd-cfDNA measurement (ml/min/1.73m2)** | 45 ±26 | 62 ± 20 | 41 ± 20 | 50 ± 26 | 0.1 |
| **eGFR at last follow-up (ml/min/1.73m2)** | 47 ± 23 | 57 ± 23 | 38 ± 21 | 54 ± 39 | 0.22 |
| **Urine Protein/Creatinine at ddcfDNA measurement** | 0.2 (IQR:0.1-0.39) | 0.1 (IQR:0.1-0.9) | 0.79 (IQR: 0.22-1.55) | 1.35 (IQR: 0.27-3.37) | **0.01** |
| **Urine Protein/Creatinine at last-follow-up** | 0.2 (IQR:0.1-1.35) | 0.2 (IQR:0.1-1.12) | 0.57 (IQR:0.15-5.6) | 1.07 (IQR: 0.45-2.4) | 0.12 |
| **Calculated panel reactive antibody** |  |  |  |  | 0.53 |
| <20% | 22 (91.7%) | 11 (91.7%) | 11 (91.7%) | 5 (83.3%) |  |
| 20-50% | 0 (0%) | 0 (0%) | 1 (8.3%) | 0 (0%) |  |
| >50% | 2 (8.3%) | 1 (8.3%) | 0 (0%) | 1 (16.7%) |  |
| ***dd-cfDNA and dnDSAs characteristics*** | | | | | |
| **dd-cfDNA level (%)** | 0.2  (IQR:0.15-0.32) | 2.55  (IQR:1.75-4.07) | 0.27  (IQR: 0.18-0.68) | 2.35  (IQR: 1.77-3.72) | **<0.001** |
| **Patients with dd-cfDNA>1% (n, %)** | 2 (8.3%) | 11 (91.7%%) | 2 (16.7%) | 6 (100%) | **<0.001** |
| **Patients with dd-cfDNA>0.5% (n, %)** | 3 (12.5%) | 12 (100%) | 4 (33.3%) | 6 (100%) | **<0.001** |
| **dd-cfDNA level and dnDSA, n(%)** |  |  |  |  | **0.001** |
| dd-cfDNA level ≥ 1% and DSAs | 1 (4.2%) | 10 (83.3%) | 0 (0%) | 5 (83.3%) |  |
| dd-cfDNA level ≥ 1% and no DSAs | 1 (4.2%) | 1 (8.3%) | 2 (16.7%) | 1 (16.7%) |  |
| dd-cfDNA level <1% and DSAs | 2 (8.3%) | 1 (8.3%) | 0 (0%) | 0 (0%) |  |
| dd-cfDNA level <1% and no DSAs | 20 (83.3%) | 0 (0%) | 10 (83.3%) | 0 (0%) |  |
| **Patients with dnDSAs, n (%)** |  |  |  |  | **<0.001** |
| No dnDSAs | 21 (87.5%) | 1 (8.3%) | 12 (100%) | 0 (0%) |  |
| Class I dnDSAs | 0 (0%) | 0 (0%) | 0 (0%) | 0 (0%) |  |
| Class II dnDSAs | 3 (12.5%) | 11 (91.7%) | 0 (0%) | 3 (50%) |  |
| Class I +II dnDSAs | 0 (0%) | 0 (0%) | 0 (0%) | 3 (50%) |  |
| **dnDSA titer, n (%)** |  |  |  |  | **<0.001** |
| Negative | 21 (87.5%) | 1 (8.3%) | 12 (100%) | 0 (0%) |  |
| <2500 | 0 (0%) | 1 (8.3%) | 0 (0%) | 0 (0%) |  |
| 2500-10000 | 0 (0%) | 5 (41.7%) | 0 (0%) | 3 (50%) |  |
| >10000 | 3 (12.5%) | 5 (41.7%) | 0 (0%) | 3 (50%) |  |

Abbreviations: dd-cfDNA, donor-derived cell-free DNA; dnDSA, de novo donor-specific antibody; MFI, mean fluorescence intensity; ABMR, antibody-mediated rejection; TCMR, T-cell mediated rejection, Tx, transplant.

**Supplemental Table 3. Binary logistic regression analysis regarding variables associated with antibody-mediated rejection**

| **Variable** | **Univariate analysis** | | **Multivariate analysis***  **(Model A)** | | **Multivariate analysis***  **(Model B)** | |
| --- | --- | --- | --- | --- | --- | --- |
|  | **Odds ratio**  **(95% CI)** | **p-value** | **Odds Ratio**  **(95% CI)** | **p-value** | **Odds Ratio**  **(95% CI)** | **p-value** |
| **Recipient age (for each 1 y)** | 0.98 (0.94-1.016) | 0.27 | 1.01 (0.92-1.08) | 0.98 | 1.05 (0.97-1.13) | 0.22 |
| **Recipient gender (female vs. male)** | 1.00 (0.31-3.19) | >0.99 | - | - | - | - |
| **Recipient race (other vs. Caucasian)** | 1.00 (0.31-3.1) | >0.99 | - | **-** | **-** | **-** |
| **Type of transplant (cadaveric vs. living)** | 0.25 (0.06-0.96) | 0.04 | 0.3 (0.015-5.95) | 0.43 | 0.43 (0.02-7.46) | 0.56 |
| **Time from Tx to dd-cfDNA measurement (for each 1 year)** | 1.25 (1.029-1.53) | **0.025** | 1.51 (0.96-2.39) | 0.07 | 1.37 (0.95-1.98) | 0.08 |
| **Serum creatinine (for each 1 mg/dl)** | 0.28 (0.08-0.88) | **0.03** | 0.14 (0.003-6.53) | 0.31 | 0.17 (0.01-3.32) | 0.24 |
| **Urine protein/creatinine ratio (for each 1 g/g)** | 1.08 (0.79-1.46) | 0.61 | - | - | - | - |
| **dd-cfDNA level (high vs. low)** | 136 (14.06-1314) | **<0.001** | **118 (5.52-2551)** | **0.002** | - | - |
| **dnDSA category**  **(DSA MFI ≥2500vs. negative DSAs)** | 88 (13.34-580) | **<0.001** | - | - | **59.4 (4.67-755)** | **0.002** |
| **Calculated Panel Reactive Antibody (>50%vs. <20%)** | 2.06 (0.26-16) | 0.48 | - | - | - | - |
| **Induction IS (ATG vs. Basiliximab)** | 1.66 (0.29-9.3) | 0.56 | - | - | - | - |
| **FK level (for each 1 ng/ml)** | 0.75 (0.57-0.98) | 0.04 | 0.57 (0.26-1.23) | 0.15 | 0.6 (0.29-1.22) | 0.15 |
| **Mycophenolic acid dose (for each 1 mg)** | 1.001 (0.99-1.002) | 0.35 | - | - | - | - |

**After multivariate adjustment for age, time posttransplant, type of transplant, serum creatinine, and FK level.*

Abbreviations: dd-cfDNA, donor-derived cell-free DNA; dnDSA, de novo donor-specific antibody; MFI, mean fluorescence intensity; Tx, transplant; IS, immunosuppression; ATG, antithymocyte globulin; ABMR, antibody-mediated rejection; TCMR, T-cell mediated rejection.
